# Therapeutic regulation of complement in patients with renal disease – where is the promise? 

**DOI:** 10.5414/CN107220

**Published:** 2011-10-04

**Authors:** Joshua M. Thurman

**Affiliations:** Department of Medicine, University of Colorado Denver School of Medicine, Denver, CO, USA

**Keywords:** complement inhibitors, renal desease, eculizumab, glomerulonephritis

## Abstract

Numerous renal diseases are characterized by complement activation within the kidney, and several lines of evidence implicate complement activation as an important part of the pathogenesis of these diseases. Investigators have long anticipated that complement inhibitors would be important and effective therapies for renal diseases. Eculizumab is a monoclonal antibody to the complement protein C5 that has now been administered to patients with several types of renal disease. The apparent efficacy of this agent may herald a new era in the treatment of renal disease, but many questions about the optimal use of therapeutic complement inhibitors remain. Herein we review the rationale for using complement inhibitors in patients with renal disease and discuss several drugs and approaches that are currently under development.

## Introduction 

It has long been appreciated that the complement system is activated in a many types of renal disease. More than 30 y ago biopsy studies of patients with immune-complex glomerulonephritis demonstrated complement activation in the glomeruli [1]. Biopsy tissue is now routinely examined for deposits of complement components by immunofluorescence microscopy. More recently it has become clear that the complement system can be activated in the kidney by many different mechanisms, and in several intra-renal locations. The complement system is an important part of the innate immune system. It plays a critical role in the elimination of pathogens, and complement activation fragments may help tissue recovery in some settings [2]. Uncontrolled complement activation can cause severe injury to self-tissue, however, and several lines of evidence implicate complement activation as a cause of injury in kidney disease. 

Given the strong evidence that complement activation is pathogenic in a variety of renal diseases, investigators have long anticipated that complement inhibitors would be important and effective therapies for these diseases [3]. Eculizumab is a monoclonal antibody to the complement protein C5. It was initially approved for the treatment of paroxysmal nocturnal hemoglobinuria [4, 5], but this drug has now been administered to patients with several types of renal disease [6, 7, 8, 9, 10, 11, 12]. The apparent efficacy of this agent may herald a new era in the treatment of renal disease, but many questions about the optimal use of therapeutic complement inhibitors remain. Which diseases are most likely to benefit from such agents? Should the complement cascades be blocked at different levels in different diseases? Many of the diseases in question are chronic, and the optimal duration of complement inhibition in these diseases has also not been established. Along the same lines, good non-invasive biomarkers of complement activation have not yet been developed. There is reason to think, therefore, that our ability to identify appropriate patients and monitor their response to complement inhibition will improve in the years ahead. 

## The complement system 

**Figure 1.**
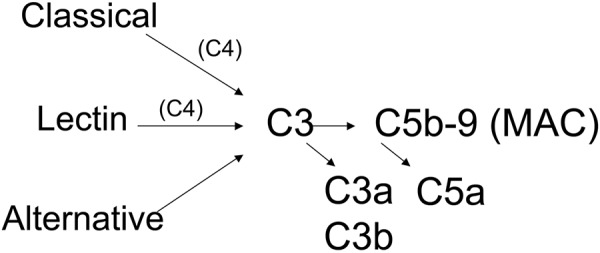
Simplified overview of the complement cascade. The complement cascade is comprised of more than 30 proteins. The cascade can be activated through three distinct pathways. C3 and C4 are routinely measured in patients suspected of having glomerular disease. C3 is the central component of the cascade, and activation of all three pathways can lead to C3 cleavage. Activation of the classical and mannose binding lectin pathway causes cleavage of C4. Activation through all three pathways generates the same downstream pro-inflammatory fragments: C3a, C5a, C3b, and C5b-9.

The complement system is an ancient part of the innate immune system and provides an important line of defense against bacterial, viral, and fungal pathogens [13]. It is a cascade of proteins, analogous to the clotting cascade, that can be activated very rapidly. The complement system has three distinct pathways by which it can be activated (Figure 1): the classical, mannose-binding lectin, and alternative pathways. Although activated by distinct molecular interactions, all three pathways have the potential to generate the same downstream effector molecules. The inflammatory effects of the complement system are primarily mediated by C3a, C5a, C3b, and C5b-9 (sometimes called the membrane attack complex (MAC) or the terminal complement complex (TCC)). Biologic effects of fragments generated from other components of the system have also been reported. C3a and C5a are soluble molecules that serve as ligands for receptors. C3b can be covalently bound to cell and tissue surfaces and is also a ligand for cognate receptors. C5b-9 is a multimeric complex that can form membrane pores. The size of the pores is variable, but they are often ~ 10 nm in diameter. The pores can cause cell lysis, or in sublytic quantities C5b-9 can cause cell activation [14]. 

Like all components of the immune system, a key function of the complement system is to discriminate self from non-self. This function is achieved in several ways. The classical pathway of complement is activated by immunoglobulin when bound to a target antigen. This activation is, therefore, as specific as that of the antibody in question. Mannose binding lectins can bind to sugar moieties expressed on the surface of bacteria [15]. Like the toll-like receptors, this arm of the complement system is activated in response to conserved molecular patterns on common pathogens. The classical and MBL pathways secondarily engage the alternative pathway. Consequently, the alternative pathway can substantially contribute to tissue injury, even when activation initially occurs through the classical or MBL pathways [16]. 

The alternative pathway is continually activated in serum, and can cause spontaneous injury on cells when it is not adequately controlled on the cell surface. Impaired control of this pathway permits it to self-activate, and uncontrolled alternative pathway activation contributes to tissue inflammation in many diseases [17]. A large number of mutations and polymorphisms in the complement control proteins are associated with defective complement regulation. Complement regulation can also be disrupted by autoantibodies to complement system proteins. Intriguingly, the kidney is particularly susceptible to injury in patients who carry systemic abnormalities in alternative pathway regulation. Defects in the function of the circulating alternative pathway regulator factor H, for example, are causally associated with atypical hemolytic uremic syndrome (aHUS), dense deposit disease (DDD), and severe cases of other renal diseases [18]. 

## Mechanisms of complement activation in the kidney 

Analysis of human samples and animal models indicates that all three activation pathways are involved in various renal diseases. 

### Classical pathway

Antibodies and immune-complexes can activate the classical pathway of complement. This system is, therefore, frequently activated in immune-complex and antibody mediated diseases. The protein C4 is a component of this system, and tissue deposits of the C4 fragment C4d are detected in the biopsies of patients with antibody-mediated diseases. Indeed, the detection of C4d has now been integrated into the diagnostic criteria for humoral allograft rejection [19]. In patients with antibody-mediated rejection, the staining of peritubular C4d likely represents activation of the classical pathway by antibodies that have bound to endothelial antigens. 

### MBL pathway

Work in recent years has revealed that the MBL pathway is activated in several renal diseases, including IgA nephropathy [20] and in selected cases of other forms of glomerulonephritis [21]. IgA may directly activate the MBL pathway [22], or MBL could possibly bind neo-epitopes that are generated or exposed within the injured kidney [23]. 

### Alternative pathway

The alternative pathway amplification loop may be engaged in diseases in which the classical and MBL pathways are activated. This pathway is also a primary cause of vascular and glomerular injury in aHUS and DDD. Surprisingly, experiments in animal models also suggest that the alterative pathway contributes to injury in “antineutrophil cytoplasmic autoantibody (ANCA)” associated vasculitis and FSGS [24, 25]. Alternative pathway activation causes tubular injury in chronic proteinuric renal diseases [26] and in acute ischemic injury [27]. 

### Complement activation as a primary or secondary phenomenon

It may be useful to think of complement as a primary or secondary immune factor in kidney diseases. In DDD, for example, dysregulated control of the complement system seems to directly injure the kidney. In an antibody-mediated disease such as lupus nephritis, complement acts downstream of immune-complexes to cause renal injury. Categorizing diseases like this may help assess whether complement inhibition should be a first line drug, or rather used as adjunct therapy for patients already receiving B and T cell targeted therapies. Unfortunately such distinctions are not absolute. DDD, for example, is not an immune-complex mediated disease, but antibodies (C3 nephritic factor) can play a role in its development. 

## Evidence that complement is pathogenic in kidney diseases 

There is evidence that complement activation contributes to the pathogenesis of a wide range of different renal diseases (Table 1). Because of the important role that the complement system plays in both innate and adaptive immune responses, this system is engaged by multiple different molecular processes within the kidney. Serologic evidence of complement activation and renal biopsy studies indicate that complement activation is associated with disease activity. A wide range of animal models has shown that complement activation plays a pathogenic role in the development of renal injury. There are numerous experiments demonstrating that complement activation contributes to the pathogenesis of immune-complex mediated renal diseases such as membranoproliferative glomerulonephritis (mpgn) Type I and membranous disease [28, 29]. More recently, animal models using targeted deletion of complement proteins or complement inhibitors have indicated a role for the complement system in unexpected diseases including renal ischemia/reperfusion injury [27], focal segmental glomerulosclerosis [25], and ANCA associated renal disease [24]. The best evidence for a pathologic role of complement activation in human disease is provided, of course, by successful treatment of the disease with complement inhibitors. A number of case reports have been published [9, 10, 11, 12], and several clinical trials are currently underway. 

Because complement activation generates several pro-inflammatory products, the mechanisms by which it causes injury can be distinct in different locations in different diseases. There are animal studies that demonstrate specific disease-inducing roles for C3a [30, 31], C5a [31, 32], and C5b-9 [28, 33]. If it were determined that particular renal diseases were caused by specific complement factors, then therapies could be focused on the pathologic factor(s). Therapies with a narrower range of action within the complement system might have fewer side-effects. 

## Biomarkers of complement activation in renal diseases 

An important and difficult problem is how to determine whether the complement system is activated in a given patient. Activation of the complement system can be inferred from several clinical findings, including the deposition of complement proteins within the kidney, perturbations of the levels of circulating C3 and C4 during disease flares [34], detection of complement activation fragments (e.g. C3a, C3d, Bb, C5a, sC5b-9) in the plasma or urine, and the association of mutations and polymorphisms in complement proteins with the development of disease. 

Although not commonly performed in clinical practice, detection of the complement activation fragments can be a useful method of detecting intra-renal complement activation. In lupus nephritis, for example, the measurement of C3a may be a more sensitive marker of disease flares than C3 levels [35] and may be predictive of disease flares [36]. Elevations in circulating C3a may also be seen in IgA nephropathy, a disease in which perturbations in intact C3 are not usually seen [37, 38]. Similarly, C3dg and C5b-9 can be detected in the urine of patients with membranous nephropathy, another disease in which levels of circulating C3 are not typically depressed [39]. Soluble C5b-9 has been used as an indicator of complement activation in aHUS, and can also be used to monitor the response to eculizumab [9]. 

Atypical HUS and DDD are fairly “pure” complement-mediated diseases, and are diseases for which there is a strong rationale in support of complement inhibition. Even in these diseases, however, there are many practical difficulties in the identification and monitoring of appropriate patients to treat. Consequently, new biomarkers would be of great benefit. Accurate biomarkers of complement activation would permit the clinician to identify patients most likely to benefit from a therapeutic complement inhibitor, and could also be used to monitor patients for relapse. 

### Atypical HUS

Atypical HUS is the renal disease with the most extensive experience using eculizumab [6, 7, 8, 10, 11, 12, 40, 41]. Atypical HUS is associated with genetic abnormalities in up to 60% of patients [42]. Complement activation may also cause tissue injury in other forms of thrombotic microangiopathy (TMA), including Shiga-toxin associated disease (typical HUS) [43]. In practice, however, the diagnosis of a TMA can be delayed or unclear, and distinguishing aHUS from other forms of TMA can take weeks. Alternative diagnoses, such as thrombocytopenic thrombotic purpura or disseminated intravascular coagulation, can be difficult to exclude in a timely fashion. Furthermore, finding objective evidence of pathologic complement activation can be exceedingly difficult. C3 levels are commonly measured in patients with suspected glomerular disease. But even in patients with aHUS and mutations in factor H, in whom complement activation almost certainly contributes to disease, C3 levels are depressed in only about 50% of the patients [44]. New functional tests may help identify patients with complement dysregulation more rapidly [45]. In my own experience, however, plasma exchange is frequently initiated before the question of complement inhibition is even raised, further complicating clinical assessment of complement activity. 

### Dense deposit disease

Like aHUS, DDD is commonly associated with defective complement regulation [46], and a clinical trial with eculizumab is currently underway (clintrials.gov NCT01221181). The diagnosis of DDD is made by renal biopsy and virtually all biopsies show C3 deposition [47]. There is, therefore, less diagnostic uncertainty than there is with aHUS since patients with suspected TMA are usually not biopsied. There are currently no clinically proven effective therapies for DDD. Approximately 50% of DDD patients reach end-stage renal disease within 10 years, and ~ 50% of renal transplants are lost within 5 years [46]. On the other hand, the rate of progression occurs over months to years, and some patients have normal renal function at the time of diagnosis. Thus, although the prognosis for patients with this disease is poor, it can be difficult to assess whether the clinical benefits justify long-term complement inhibition. 

### Lupus nephritis

Like DDD, the definitive diagnosis of lupus nephritis is usually made only after a renal biopsy has been performed. Most patients with proliferative lupus nephritis undergo induction therapy with cyclophosphamide or mycophenolate mofetil based therapies [48, 49, 50, 51]. Experience with rituximab shows that it may be difficult to demonstrate in a randomized clinical trial a benefit to adding-on an additional agent to the standard regimens [52]. On the other hand, in some studies up to 50% of patients do not respond to standard induction therapies [48, 51]. Direct complement inhibitors would block an effector system that is not directly affected by MMF or cyclophosphamide, and add to the effective scope of B-cell and T-cell targeted therapies. Furthermore, the response to standard induction therapies can take months [48, 53], and complement inhibitors could theoretically suppress glomerular inflammation almost immediately. Thus, although the role for therapeutic complement inhibitors in patients with lupus nephritis remains to be defined, these agents may fill an unmet need in the treatment of this disease. 

### The ideal biomarker

The requirements for a complement biomarker vary from disease to disease. In a disease like aHUS a sensitive systemic marker of complement activation, particularly one not confounded by plasmapheresis, would be critically useful. In DDD, the non-invasive detection of glomerular C3 deposition could help guide treatment with a complement inhibitor. In a disease like lupus nephritis that has established therapies, the detection of intra-renal complement activation would be invaluable determining whether the addition of a complement inhibitor is likely to add benefit to the agents the patient is already receiving. 

My laboratory has developed an MRI-based method for the detection of intra-renal complement activation [54]. This method employs a contrast agent that is targeted to tissue-bound C3d and is then detected by T2-weighted MRI. Our hope is that this could provide a global picture of inflammation, and specifically complement activation, throughout both kidneys. The bound C3 fragments are a useful and robust marker of disease activity insofar as they are abundant and they are durably fixed to tissues. It is worth noting, however, that the detection of C3 cleavage may not be useful in the context of treatment with eculizumab, a drug that blocks the complement system at the level of C5. 

## Therapeutic options 

A large number of agents have been developed, or are currently being developed, for the therapeutic inhibition of the complement system [55]. Eculizumab is a monoclonal antibody to C5 that prevents the generation of C5a and C5b-9 [41]. Eculizumab has now been used in a large number of patients with complement-dependent renal disease [6, 7, 8, 9, 10, 11, 12, 40], and several clinical trials of this agent in these diseases are currently underway. Purified Serping1 (a circulating inhibitor of C1) is approved for the treatment of hereditary angioedema, a disease caused by deficiency of the protein [56]. Reports also suggest that administration of this protein may reduce tissue injury in other diseases [57, 58, 59]. 

There are other clinically available drugs also known to have complement modulating activity, including IVIg [60] and heparin [61]. Given the wide clinical experience with a drug like heparin, a greater understanding of its ability to modulate complement activation could potentially give it a role in treating inflammatory diseases. In light of evidence that there are interactions between the complement system and the clotting cascades, a drug that inhibits both systems may be particularly useful for diseases such as the TMAs [62]. Plasmapheresis can modulate complement activation in several ways. It can remove activating antibodies and immune-complexes. Replacement of the removed patient plasma with fresh frozen donor plasma can also restore circulating inhibitory proteins, such as factor H. This is a presumed mechanism by which plasma exchange benefits patients with deficient factor H function in aHUS and DDD. 

A large number of other complement inhibitory agents are in development, including recombinant constructs made from endogenous complement regulatory proteins, monoclonal antibodies to specific complement components or receptors, small molecules with complement inhibitory activity, and peptide inhibitors of the C3a and C5a receptors [55, 63, 64]. Although a full discussion of the agents under development is beyond the scope of this review, the features of some of these agents are noteworthy. Monoclonal antibodies to components of activation pathways can selectively block activation through a single pathway. For example, antibodies to factor D and factor B that inhibit activation through the alternative pathway have been developed [63, 65]. These agents may be useful in diseases, such as aHUS and DDD, where complement activation appears to be dependent upon this pathway. A small peptide inhibitor of C3 activation (POT-4) and a cyclic hexapeptide inhibitor of the C5a receptor (PMX-53) are currently being tested in clinical trials. These drugs offer the possibility of blocking complement activation at an earlier stage (e.g. POT-4), blocking a specific complement pathway, or selectively blocking one activation fragment (e.g. PMX-53). Agents with a more selective mechanism of action may have improved efficacy in a given disease with a reduced risk of side-effects. 

Several agents have also been developed to target therapeutic complement inhibitors to specific tissue sites. A recombinant form of complement receptor 1 (CR1) was one of the first agents developed as a therapeutic complement inhibitor [66]. An agent called APT070 links the complement regulating region of CR1 to a peptide that associates with cell membranes, and with a myristoyl group that inserts into the cell membrane [67]. Experiments in animal models indicate that allografts treated with this agent are protected from ischemic and immune-mediated injury [67, 68]. Tomlinson and colleagues have developed several complement receptor 2 (CR2)-targeted complement inhibitors [69, 70]. These agents bind at sites of complement activation (tissue-bound iC3b/C3d), conferring local complement inhibition without long lasting systemic inhibition. These agents appear to have superior pharmacokinetics to untargeted inhibitors, and they may also pose less infectious risk [71]. Another targeted inhibitor was developed using an antibody that binds an epitope expressed in the rat proximal tubule. This complement inhibitor protected rats from tubular injury in puromycin induced nephrosis [72]. Further development of complement inhibitors targeted to sites of complement activation or to specific tissues should improve the efficacy and pharmacokinetics of these agents while also reducing their potential toxicity. Such improvements will be a great advantage, particularly for patients who require chronic treatment. 

## Issues that may affect the efficacy of complement inhibitors 

### Half-life and tissue penetration

Depending upon their mechanism of action, a complement inhibitor may or may not require access to the site of complement activation. Agents that work by inactivating the C3 and/or C5 convertases, that block complement receptors, or that are targeted to tissue sites will obviously need adequate tissue penetration. There is also evidence that tubular epithelial cells can locally produce complement components, contributing to tissue injury [73, 74, 75]. Complement inhibitors used to treat tubular injury may, therefore, need to penetrate the tubulointerstitium. Tissue inflammation increases local vascular permeability, of course, and glomerular perm-selectivity is increased in many glomerular diseases. Nevertheless, for some complement inhibitors the ability of the molecule to access the site of injury will need to be verified. 

### Complement inhibitors in patients with genetic mutations or with activating autoantibodies

As described above, uncontrolled pathologic complement activation in patients with aHUS and DDD can be caused by a number of defects. Loss of function mutations in inhibitory proteins can have the same physiologic effect as gain of function mutations in activating proteins. Furthermore, the loss of complement control may be due to auto-antibodies to complement regulatory proteins such as factor H [42]. This wide range of molecular defects raises the possibility that some complement inhibitors will be less effective in some particular patients. For example, agents that work at the level of the C3 convertase may be less effective in a patient with a gain of function mutation in the *C3* gene. Similarly, agents that restore native or recombinant forms of factor H may be less effective in patients with auto-antibodies to factor H or who have C3 nephritic factor. Many nephrologists do not have access to sophisticated complement testing. Even if such testing is available, it can take months to identify complement gene mutations. Because eculizumab targets a downstream component of the complement cascade it should still be effective at blocking C5a and C5b-9 generation in patients with the various described defects. As new agents enter clinical use, though, the possibility that the agent may not work in selected patients must be kept in mind. 

### Risk of infection with therapeutic complement inhibitors

The complement system has been shown to play a role in a wide range of physiologic functions, including defense against infection, removal of apoptotic cells, immune-complex clearance, tissue repair, and coagulation [62]. Given the well-described role of complement in the innate and adaptive immune responses, infection is the most obvious complication of a therapeutic complement inhibitor. An increased risk of infection in patients treated with a complement inhibitor may be inferred from the increased risk seen in patients with congenital complement deficiencies. Patients with deficiencies in classical pathway proteins, alternative pathway proteins, regulatory proteins, and proteins of the terminal complement complex have been identified [62]. The susceptibility of patients to particular organisms is related to where the defect is in the complement cascade. It is also worth noting that deficiencies in complement regulatory proteins also increase the risk of infection due to the consumption of factors such as C3 [76]. Thus, patients with aHUS or DDD may have an increased risk of infection prior treatment. 

Overall, infection with Neisseria meningitidis appears to be the most common infectious complication of complement deficiency [77], and this is by far the most common infection in those with deficiencies in terminal complement proteins (i.e. C5 – C9) [78]. Eculizumab (which blocks complement activation at the level of C5) carries a black box warning that patients should be immunized against Meningococcal infections at least two weeks prior to treatment with the drug. The most extensive experience with this drug is in patients with PNH, three patients who had been immunized against *N Meningitides* developed Meningococcal sepsis while being treated for PNH [79]. Thus, although the risk of infection in patients treated with complement inhibitors may be mitigated, caution is clearly warranted. 

## The future 

Pathogenic complement activation contributes to a wide range of renal diseases. Eculizumab has now been used in dozens of patients with aHUS, anti-phospholipid antibody syndrome, DDD, or at risk for humoral allograft rejection. As experience with this drug grows it will undoubtly be used in patients with other types of renal disease too. The involvement of the complement system in renal disease is complex. There are multiple mechanisms of complement activation, and activation generates multiple biologically active fragments. Within this complexity lies opportunity. In recent years there has been much work exploring the role of specific complement components in causing tissue injury or in facilitating tissue repair. Furthermore, the therapeutic complement inhibitors under development will give clinicians the ability to block specific pathways or complement components. 

Nephrologists often have direct evidence of abnormal complement activation in an individual patient. This may be an important aspect of the clinical use of complement inhibitors, and it distinguishes these agents from many of the other new biologic therapies. The development of new biomarkers and clinical testing should further improve our ability to detect and characterize complement activation in patients. New methods may thus allow clinicians to identify the pathways that are engaged in a patient, localize complement activation within a given organ, and choose therapeutic agents designed to block specific complement components at that specific location. Furthermore, the dosing and duration of treatment could be tailored to a patient’s response. The use of eculizumab in patients with renal disease over the past few years has been an important advance in the care of patients with renal disease. Given all the new approaches and therapeutics under development, however, anti-complement therapies may well emerge in years to come as one of the great success stories of individualized medicine. 

## Acknowledgment 

This study was supported by NIH grant DK076690. The author is a consultant for Alexion Pharmaceuticals, Inc. 

**Table 1.** Table 1.

Syndrome	Disease	Systemic levels	Biopsy	Genetic association (selected references)	Animal model (selected references)
Nephritic syndrome	Lupus nephritis	✓	✓	✓ [80]	✓ [16, 83]
	MPGN I	✓	✓	✓ [81]	✓ [28]
	MPGN II (DDD)	✓	✓	✓ [82]	✓ [84]
	IgA Nephropathy		✓		
	ANCA associated vasculitis				✓ [24]
	Post-strep GN	✓	✓		
Nephrotic syndrome	Membranous GN		✓		✓ [29]
	FSGS		✓		✓ [25]
	Diabetic nephropathy		✓		
Tubular injury	Ischemic AKI		✓		✓ [27, 85]
	Tubular injury in proteinuric disease		✓		✓ [26]
Allograft rejection	Humoral		✓		
	Cellular				✓ [74, 86]
TMA	Atypical HUS	✓	✓	✓ [82]	✓ [89]
	TTP			✓ [87]	
	HELLP			✓ [88]	

MPGN = membranoproliferative glomerulonephritis; ANCA = anti-cytoplasmic nuclear antigen; GN = glomerulonephritis; FSGS = focal segmental glomerulosclerosis; AKI = acute kidney injury; TMA = thrombotic microangiopathy; TTP = thrombotic thrombocytopenic purpura; HELLP = syndrome of hemolysis, elevated liver enzymes, and low platelet count.
